# Sustainable Insect-Based Diets in Sub-Saharan Africa: A Review of Prevalence, Acceptability and Impact on Nutritional Status

**DOI:** 10.3390/nu18091414

**Published:** 2026-04-29

**Authors:** Maria Rouco, Charity Chinonso Ugwu, Gabriel Reina, Silvia Carlos

**Affiliations:** 1 School of Pharmacy, Universidad de Navarra, 31008 Pamplona, Spain; mrouco.1@alumni.unav.es; 2 Faculty of Pharmaceutical Sciences, Nnamdi Azikiwe University, PMB 5025, Awka 420110, Anambra State, Nigeria; ugwuchinonso93@gmail.com; 3 Microbiology Department, Clínica Universidad de Navarra, 31008 Pamplona, Spain; 4 Navarra Institute for Health Research (IdiSNA), 31008 Pamplona, Spain; scarlos@unav.es; 5 Department of Preventive Medicine and Public Health, Universidad de Navarra, 31008 Pamplona, Spain

**Keywords:** insects, diet, acceptability, nutrition, sustainable, sub-Saharan Africa

## Abstract

Malnutrition—including undernutrition, micronutrient deficiencies and overweight—remains a major public health concern in sub-Saharan Africa, largely driven by food insecurity. Edible insects have been proposed as a sustainable, nutrient-dense dietary alternative with potential to improve food security and nutritional outcomes. This review analyses studies published until January 2024 in PubMed and Google Scholar assessing the prevalence, acceptability and nutritional impact of insect-based diets in sub-Saharan Africa. Thirteen original studies, predominantly qualitative, conducted in 8 of 47 countries in the region, met inclusion criteria. Two reviews provided additional evidence. Most studies focused on acceptability, which was strongly influenced by cultural and religious norms. Higher acceptance was observed among older individuals and those with lower educational attainment, while younger and more urbanized populations showed greater reluctance. Reported motivations for consumption included tradition, taste and perceived nutritional value. Some studies highlighted potential health risks related to food safety and the need for improved regulatory frameworks. The available nutritional analyses showed that edible insects are rich in protein and essential micronutrients, particularly iron and zinc, suggesting their potential to address common deficiencies. Although evidence on long-term nutritional impact remains limited, current findings support the feasibility and potential public health relevance of promoting insect-based diets in low-income settings.

## 1. Introduction

Malnutrition, as defined by the World Health Organization (WHO), encompasses deficiencies, excesses, or imbalances in a person’s intake of energy and/or nutrients [1]. Undernutrition is a major contributor to child mortality, being associated with approximately half of all deaths among children under five years of age [1,2,3]. The burden is disproportionately concentrated in low- and middle-income countries, particularly in sub-Saharan Africa (SSA), where poverty significantly exacerbates the risk [2,3]. Moreover, malnutrition increases healthcare costs, reduces productivity, and hinders economic development, thereby perpetuating a cycle of poverty and poor health outcomes [2].

According to the 2025 estimates from the UNICEF/WHO/World Bank Joint Child Malnutrition Estimates, 150.2 million children under five years of age worldwide were affected by stunting, 42.8 million by wasting, and 35.5 million by overweight. Although global stunting has been consistently declining since 2000, progress in Africa has been limited, with 43% of children affected by severe stunting residing on the continent [3].

Access to nutritionally adequate foods—such as fruits, vegetables, legumes, fish, meat, and dairy products—remains a challenge for many households. In contrast, energy-dense foods with poor nutritional quality, high in saturated and trans fats, added sugars, and salt, are often more affordable and readily available. This imbalance contributes to the coexistence of multiple forms of malnutrition, known as the triple burden of malnutrition, which includes undernutrition, micronutrient deficiencies, and overweight or obesity. This phenomenon represents a growing public health concern worldwide, particularly in SSA [1,4].

A key underlying driver of malnutrition is food insecurity. The Food and Agriculture Organization of the United Nations (FAO) defines food insecurity as the lack of regular access to sufficient safe and nutritious food necessary for the normal growth, development and maintenance of an active and healthy life. This inadequacy results from either the unavailability of food and/or the insufficient resources to obtain food [5]. Food insecurity is expected to intensify as the global population continues to grow—projected to reach approximately nine billion by 2035—highlighting the need for innovative and sustainable food production strategies [6]. Particularly, the African population that is facing severe food insecurity corresponds in number with those suffering from undernutrition [5].

Preventing malnutrition requires a multifaceted approach, including adequate maternal nutrition before and during pregnancy and breastfeeding, optimal infant feeding practices, and access to diverse, safe, and nutrient-rich foods during early childhood. Additionally, a healthy environment—characterized by access to healthcare, clean water, sanitation, hygiene, and opportunities for physical activity—is essential [7]. However, addressing malnutrition remains complex due to intersecting factors such as population growth, poverty, armed conflict, climate change, and the long-term impacts of the COVID-19 pandemic [3].

To address malnutrition, different alternative diets have been proposed over the years. These alternatives need to meet some specific requirements to be considered suitable, including high nutrient contents (i.e., high-quality proteins), health-promoting properties, minimal or no adverse effects, high sustainability and consumer acceptance [8]. Strategies such as bio-fortification and the promotion of underutilized crops rich in nutrients have been identified as promising strategies to enhance dietary diversity and food security [9]. Among these, entomophagy—the consumption of insects—has gained increasing attention.

In recent years, edible insects have been recognized as a potentially sustainable and nutrient-dense food source. While their consumption remains uncommon in most European and North American countries, entomophagy is widely practiced in many parts of the world, such as sub-Saharan countries. It is estimated that more than 2 billion people globally include insects in their regular diets [10]. It is an indigenous practice that has a significant role in the tradition and religion of a wide range of cultures and regions since ancient times. There is evidence of the consumption of insects as a traditional dish in diverse regions of China, Thailand, Japan, Latin America and Africa [11]. However, there is still not much evidence available from SSA [10].

Insects could help in the triple-burden of malnutrition due to their variable composition in nutrients such as protein, lipids and some essential micronutrients like iron, zinc or calcium. Studies supported by the FAO highlight their nutritional potential, particularly in communities where they are already traditionally consumed [12]. Furthermore, this type of diet could also be a sustainable agricultural method of nourishing the population, especially at this time when the impact of climate change is becoming more significant [13]. Edible insect farming could contribute to minimizing nutrient and energy cycling, mitigating biodiversity loss and slowing climate change [13]. Its potential to enhance the environment would be interesting due to its capacity of converting solid waste into valuable resources, such as organic matter. Moreover, insect farming requires substantially fewer natural resources than conventional livestock production, including lower land and water use (e.g., approximately 2 L of water for cricket production compared to 112 L for beef) [13,14].

From a regulatory perspective, the FAO has promoted edible insects since 2013 as an “underexplored” resource with the potential to enhance global food security [15]. The available information about safety is limited. The association with some health risks, such as allergens and chemical or biological dangers, has been reported. The level of contaminants and hazards found in these insect-based diets depends on factors such as insect species, harvest stage, feed substrate and processing methods. It is crucial to ensure effective control of these aspects for enhancing food safety [15]. Hygiene plays an essential role in minimizing toxic substances (i.e., mycotoxins, heavy metals and pesticides) and pathogens (i.e., bacteria, fungi and parasites) [13]. The European Commission regulates the consumption of insects as a novel food (Regulation (EU) 2015/2283 of the European Parliament), which ensures that the insects being marketed are safe and have undergone a scientific evaluation by the European Food Safety Authority (EFSA). Furthermore, the EFSA approved in 2023 the consumption of four insects in Europe, while approval for eight more is pending [16]. The majority of studies indicate that countries with lower consumption of edible insects tend to have stronger food policy for regulating their use, while in continents like Africa and Asia, where the tradition of entomophagy is widespread, insect regulation is weaker or nonexistent [14]. There are already some established standards in some African countries, specifically Malawi, Tanzania, Kenya and Uganda. Most of them are currently in transition stages of traditional and modernized legislation aligning with international specifications on their food security systems, such as Codex Alimentarius. However, this legislation does not accommodate the complex production chain and the socio-economic factors that influence the integration of insects in the food system [17]. The indigenous processing methods for manipulation of insects often do not meet the standards of food safety [17]. Therefore, the development of harmonized regulatory frameworks will be essential to support safe scaling, investment, and commercialization of insect-based foods, from local to industrial perspective [15].

Considering these factors, the use of edible insects as an alternative protein source in malnutrition-affected regions of SSA represents a promising strategy, given their nutritional, economic, and environmental advantages, provided that their adoption is culturally acceptable and supported by appropriate safety measures.

The aim of this study is to review scientific evidence on the prevalence, acceptability, and nutritional impact of edible insect-based diets in sub-Saharan Africa.

## 2. Methods

### 2.1. Literature Search and Search Strategy

A review was conducted to identify published studies evaluating the frequency of use of edible insects in sub-Saharan Africa, as well as the acceptability and nutritional impact. The literature review was conducted using PubMed and Google Scholar until January 2024. The terms “insect” and “food” or “diet” or “nutrition” and “agriculture” and “Africa” were included and combined in the search strategy. The references of published reviews and meta-analyses were also reviewed. Additional references that were identified in articles during screening were also manually searched.

After the general search for articles from SSA, the search was repeated by specifying each of the countries in this region: Angola, Benin, Botswana, Burkina Faso, Burundi, Cameroon, Cape Verde, Central African Republic, Chad, Comoros, Congo, Ivory Coast, Democratic Republic of Congo, Djibouti, Eritrea, Ethiopia, Gabon, Gambia, Ghana, Guinea, Guinea-Bissau, Equatorial Guinea, Kenya, Lesotho, Liberia, Madagascar, Malawi, Mali, Mauritania, Mozambique, Namibia, Niger, Nigeria, Rwanda, Sao Tome and Principe, Senegal, Sierra Leone, Somalia, South Africa, Sudan, Swazi-land, Tanzania, Togo, Uganda, Zambia, Zanzibar and Zimbabwe.

Publication language was restricted to English, Spanish, Italian, French, or Portuguese.

### 2.2. Inclusion and Exclusion Criteria

Studies were included if they met the following criteria: (1) studies involved humans; (2) assessed edible insects in sub-Saharan Africa; and (3) had full-text access.

Studies were excluded if: (1) they were written in languages other than English, Spanish, Italian, French, or Portuguese; (2) articles were study protocols.

### 2.3. Data Extraction

Two investigators (MR and SC) independently and manually evaluated eligible articles, removing duplicates. They first assessed their eligibility reviewing all titles and abstracts and final decision on inclusion was based on a full-text assessment. Discrepancies were resolved by consensus, and a third researcher (GR) was consulted if disagreements persisted.

Both reviewers (MR, SC) extracted the following information from the reviewed studies: authors, publication year, study country and period, study design and participants’ details (number, age, and sex), data collection methods and variables and main findings.

### 2.4. Study Quality Assessment

The quality of the included individual studies was assessed by two reviewers (MR and SC) using criteria adapted from STROBE and PRISMA recommendations.

## 3. Results

### 3.1. Selected Studies

**Figure 1:**
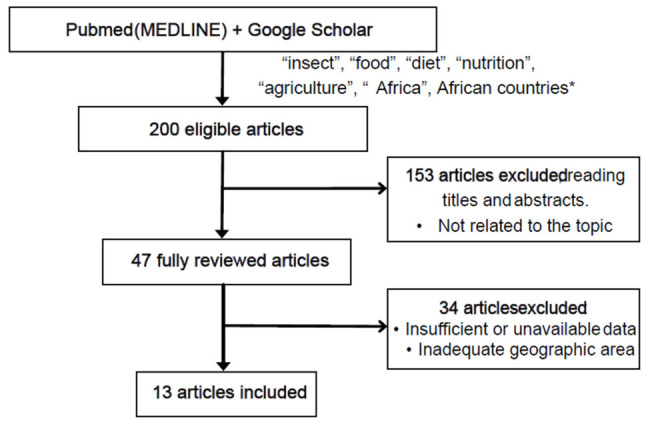
Flowchart of the studies selection. * each African country was searched as detailed above in the search strategy.

Two hundred articles were found considering the inclusion and exclusion criteria previously explained. Among all the articles, 153 were excluded after reading the titles and abstracts, obtaining a total of 47 articles. After reading the full text, 13 articles were finally selected (Figure 1). Furthermore, two related reviews were found and provided evidence, although their main objective differed from that of this review.

The majority of the selected studies are qualitative studies (62% of them). In addition, two randomized trials, one field intervention, two cross-sectional studies and one series of cases have been selected (Table 1).

**Figure 2:**
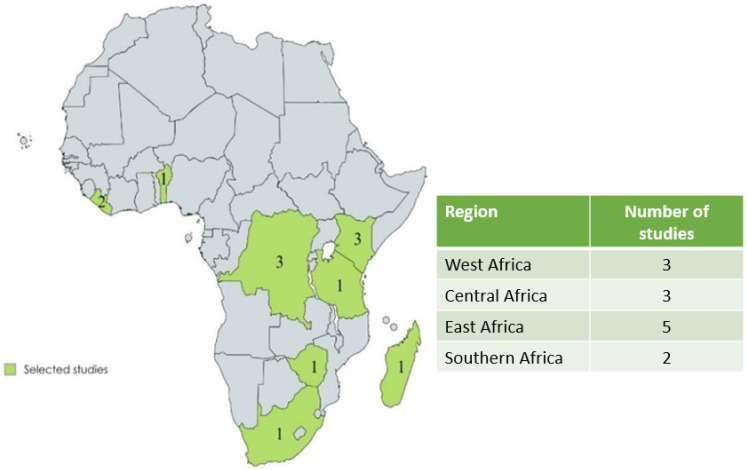
Map showing the sub-Saharan African countries where studies evaluating insect-based diets were carried out and the number of studies in each country (source: created by the author).

The studies were carried out in 8 out of the 47 (17%) SSA countries, mainly in the Democratic Republic of the Congo (DRC) (three studies), Kenya (three studies) and Liberia (two studies), with one study in the rest of the countries included: Benin, Madagascar, South Africa, Tanzania and Zimbabwe (Figure 2). All the detailed information of the selected studies is shown in Appendix A, Table A1.

Most of the thirteen selected articles had been published recently, with the least current articles published in 2017. The study period in which the studies had been carried out goes from 2014 to 2022. However, there are five articles for which the study period was not reported. In recent years, a rise in the number of publications is observed.

### 3.2. Acceptability of Edible Insect-Based Diets in Sub-Saharan Africa

Most of the results of the selected studies were related to the acceptability of edible insect-based diets.

**Figure 3:**
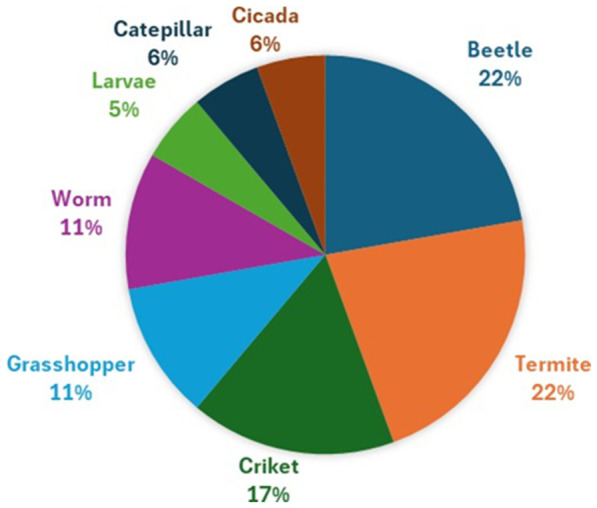
Most popular edible insects reported in sub-Saharan African studies.

The most popular edible insects mentioned in the selected articles are beetles (beetle grubs, adult beetles, *Rhynchophorus phoenicis*, *Coleoptera pupae*), termites (*Macrotermes subhyalinus*) and crickets (*Ruspolia differens*), followed by grasshoppers (Longhorn grasshopper, locust), caterpillars and cicadas. Apart from insects, worms (Mapone worms) and larvae (*A. mellifera* larvae) are also mentioned (Figure 3). The wide diversity is linked to the richness of the natural environmental conditions.

Preferences for different species are influenced by various factors including geographic location, seasonality, culture, taste, shape, size, color and perceived nutritional value.

The acceptability of insect-based diets is influenced by several factors, including religious beliefs. While certain religions, such as Islam, may restrict or discourage the consumption of specific insects, others regard them as nutritious and beneficial for health. These perceptions are closely linked to cultural traditions, particularly among indigenous communities. In some contexts, insects are viewed as unclean or unappealing, and their consumption is associated with a perceived risk of illness, including symptoms such as vomiting and abdominal discomfort [6,23,26].

The selected studies do not reflect an abundance of symptoms or health risks associated with their consumption. The most mentioned health risks by the studies population are throat itching, stomachache, and diarrhea, usually linked with excess consumption or preparing them improperly. Certain allergies associated with this practice are also mentioned [19,23,25,28].

**Figure 4:**
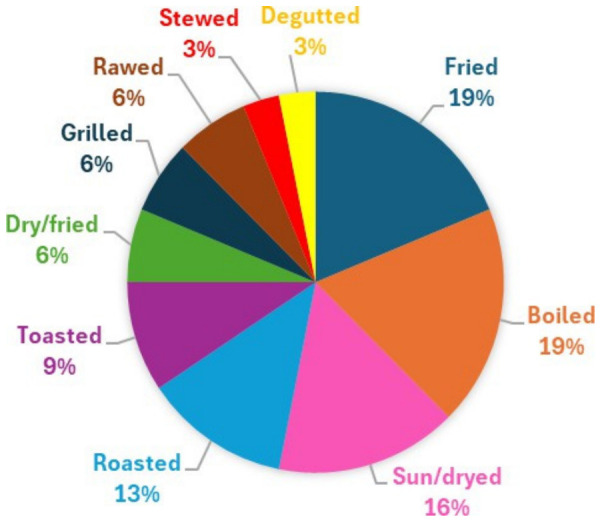
Preferred methods of processing edible insects.

The processing methods are really important for the preservation and acceptability of the raw material. The insects could be consumed directly or subject of conservation for further uses or their sale in the market [20]. The most popular methods used to process the insects are boiling and frying (19% each one), followed by sun/drying (16%) and roasting (13%). In addition, the toasting (9%), dry/frying (6%), grilling (6%), stewed (3%) and degutting (3%) techniques are also mentioned. There is even mentioned in two studies the possibility of eating them raw (6%) (Figure 4).

In general, between 70 and 90% of the population in the selected studies like to eat insects, with no notable distinction observed between men and women [18,19,23,24].

Nevertheless, the levels of consumption and tolerance are notably higher among older people (>56 years) compared to younger ones (<35 years) [23]. This increased acceptability with increasing age coincides with the transmission of the tradition, primarily by the elder generation. Nevertheless, the tradition of edible insects is also transmitted by friends, in the market, observing people collecting them or watching reports on television [18].

Some studies suggest that the acceptability of insects is also conditioned by the educational level of the population, as it plays a crucial role in fostering a positive attitude towards their consumption [21,24]. Individuals with primary and secondary education levels are often the ones dominating the value chain in insect consumption, while rich households with university education are the ones with less consumption [6,23]. This may be attributed to the fact that in richer households the ingestion of meat and fish is higher, resulting in reduced insect consumption. In contrast, poorer households have less access to meat and fish and rely more on insects as a source of protein. The greater consumption of meat and fish proteins correlates with a lower proportion of protein intake from insects [27].

### 3.3. Reasons for the Consumption of Edible Insect-Based Diets

**Figure 5:**
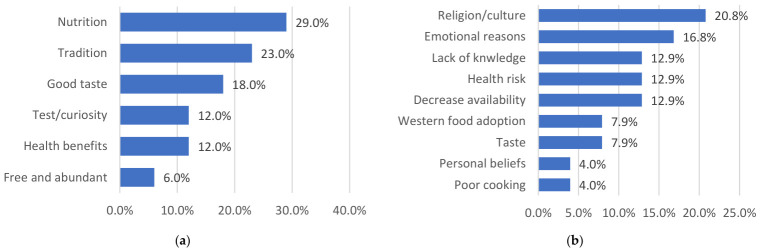
(**a**) Reasons for consuming edible insects; (**b**) reasons for the rejection of edible insects.

The motivations for consuming edible insects are multifactorial. The most frequently reported reason is their perceived nutritional benefit (29%), as insects are considered an important source of protein and may serve as a reliable food resource during periods of food insecurity. In such contexts, they function as a resilient and readily available dietary buffer. Tradition also represents a major driver (23%); in some regions, insects are regarded as delicacies and are served at social events such as weddings, where they symbolize respect and social acceptance within the community. Other commonly cited reasons include their sensory appeal, particularly taste (18%), and curiosity or willingness to try novel foods (12%). Additionally, a proportion of individuals (12%) consume insects due to beliefs in their medicinal properties, attributing benefits such as skin improvement or positive effects on infant health. A smaller segment of the population highlights their accessibility and abundance, as well as their contribution to dietary diversification (6%) (Figure 5a).

Conversely, several factors contribute to the rejection of edible insects. The most prominent barriers are religious and cultural beliefs (21%), often associated with taboos surrounding insect consumption. Emotional factors (17%), including disgust, fear of stigmatization, and psychological discomfort, also play a significant role. Limited knowledge about entomophagy (13%), concerns regarding potential health risks (13%), and the declining availability of certain insect species (13%) further discourage consumption. Moreover, processes of acculturation—particularly the increasing adoption of Western dietary patterns (8%)—have been identified as an additional influencing factor. Other less frequently reported reasons include personal beliefs (4%) and inadequate knowledge of appropriate preparation methods (4%) (Figure 5b).

The techniques used for harvesting edible insects vary according to species and seasonal availability, with most collection occurring during the rainy season (September–May). In many cases, insects are obtained from the wild through methods such as handpicking from trees or the ground, as well as light trapping [1,6,7,9]. However, these practices can be labor-intensive and increasingly challenging due to declining insect populations in several regions. For example, in Madagascar, approximately 80% of the population has reported a decrease in insect harvest quantities in recent years [27].

Insect farming represents an alternative to wild collection, although it requires substantial labor and ongoing maintenance. Evidence from selected studies suggests that farming can be relatively manageable when sufficient feed resources and adequately trained or motivated personnel are available [25,26].

In certain regions, insect production is primarily for local consumption, whereas in others it supports commercial trade, including import and export activities. For instance, Benin lacks significant domestic insect farming but participates in regional trade by importing and exporting insects to neighboring countries such as Niger and Nigeria [18]. These markets have the potential to generate profits in SSA and to improve the economy of the local communities [25,30].

### 3.4. Nutritional Impact of Edible Insect-Based Diets in Sub-Saharan Africa

Edible insects are a rich source of protein, essential amino acids, minerals, and trace elements. Their high protein content has been associated with improved bioavailability of key minerals, including iron, manganese, copper, and zinc [29].

In rural settings, where the intake of animal-derived protein is often limited, edible insects play a significant nutritional role, contributing approximately 10% of total protein consumption—comparable to the contribution of pork and beef. Nevertheless, legumes remain the primary protein source in these regions [27].

Certain species of edible caterpillars exhibit higher protein content (up to 28 g/100 g) than chicken meat (approximately 21 g/100 g). The amino acid profile varies by species but is generally characterized by high levels of lysine and threonine, alongside relatively lower concentrations of methionine and cysteine. Protein digestibility is comparable to that of casein or soy (approximately 77–98%) and may be further enhanced by removing the chitin-rich exoskeleton. For instance, the consumption of 100 g of *Imbrasia truncata* or *Imbrasia epimethea* caterpillars can provide between 30.6% and 32.3% of the recommended protein intake for a 75 kg adult [30].

In addition to protein, insects often contain higher levels of iron and calcium than conventional animal protein sources such as beef, pork, and chicken [29]. Notably, taxa such as beetles, caterpillars, ants, grasshoppers, locusts, crickets, stink bugs, termites, flies, and cockroaches are particularly rich in trace elements, and many of these species correspond to those most commonly consumed in SSA [29].

In certain regions of Madagascar, adult scarab beetles are among the preferred edible insect species. These insects provide substantial amounts of micronutrients, with reported contents of 9.1 mg of iron and 8.8 mg of zinc per 100 g portion. Such levels can contribute significantly to daily micronutrient requirements, particularly during the harvesting season (October–December) [27].

Evidence also suggests that incorporating edible insects—especially house crickets—into the diet may help alleviate zinc deficiency, particularly in vulnerable populations such as children and pregnant women in rural communities of SSA [22,29]. In addition, the inclusion of house crickets has been associated with a measurable increase in dietary energy contribution (from 19% to 23%) [22]. Caterpillars, for instance, provide an energy content comparable to pork (379 kcal/100 g vs. 416 kcal/100 g, respectively) and are rich in mono- and polyunsaturated fatty acids, particularly linolenic and linoleic acids [30].

Data on the vitamin content of edible insects remain variable across studies, likely reflecting differences in species, insect diet, and analytical methodologies. Nonetheless, current evidence suggests that a 100 g portion of insects alone is generally insufficient to meet daily vitamin requirements [30].

The integration of edible insects into diets may be facilitated through the development of enriched flours and their incorporation into commonly consumed foods, such as biscuits, bread, energy bars, cereals, cookies, and maize-based porridges. Furthermore, processing methods—including frying, boiling, roasting, sun-drying, or preparation as relishes—can enhance their sensory acceptability and overall palatability [6,11,28].

## 4. Discussion

Although the available evidence on edible insect consumption in sub-Saharan Africa remains limited, the findings synthesized in this review provide important insights into their acceptability, determinants of consumption, and nutritional potential. Beetles and crickets emerge as some of the most commonly consumed taxa [18,25,27], although preferences vary considerably according to geographic location, cultural context, and seasonal availability. Despite reported adverse effects such as throat irritation or gastrointestinal discomfort, entomophagy remains widely accepted across many communities [19,23,24,25,26,27]. Key factors influencing consumption include perceived nutritional benefits, cultural traditions, education level, and accessibility. However, significant challenges persist, particularly the declining availability of wild insect populations and the labor-intensive nature of insect farming. Despite these constraints, the development of insect-based markets presents an opportunity to support local economies in several SSA regions.

In contrast to SSA, the consumption of edible insects in high-income regions such as Europe and North America remains limited [14,31,32,33]. Although interest in insects as a sustainable protein source has increased in recent years, their adoption in Western diets is still hindered by cultural stigma and low consumer acceptance [34].

Conversely, the relatively high acceptance observed in SSA suggests that insect-based diets could represent a culturally appropriate and context-specific strategy to help address the long-standing triple burden of malnutrition in the region. In this context, ensuring food safety and promoting adequate hygiene practices are essential for facilitating their broader adoption.

Generational differences in acceptability are also evident, with higher levels of acceptance generally reported among older populations [11,18,23]. However, the increasing influence of Western dietary patterns—driven by globalization and socioeconomic changes—appears to contribute to a gradual decline in traditional insect consumption, particularly among higher-income groups with greater access to conventional animal protein sources such as meat and fish. This dietary transition may undermine the potential role of entomophagy in improving nutritional outcomes. Nevertheless, given their high acceptability and nutritional value, edible insects could play a key role in mitigating malnutrition and food insecurity, especially among vulnerable populations such as children and pregnant women.

The evidence on the nutritional impact of edible insects in SSA is still scarce [6,22,27,29]. Available studies indicate that insects are rich in essential micronutrients—including iron, calcium, and zinc—as well as high-quality proteins and amino acids [27,29]. In some cases, their protein content exceeds that of conventional livestock products. These characteristics highlight their potential to address nutrient deficiencies and contribute to improved food security in low-resource settings. Furthermore, the incorporation of insects into diverse food products and culinary preparations may enhance both their acceptability and nutritional contribution. Nonetheless, further research is required to better characterize species-specific nutritional profiles, bioavailability, and long-term health effects.

The sustainability of edible insect-based diets in sub-Saharan Africa (SSA) should be considered through environmental, economic, and social dimensions. From an environmental perspective, insects offer clear advantages over conventional livestock, including lower land, water, and feed requirements, as well as reduced greenhouse gas emissions [13,14]. Their capacity to convert organic waste into valuable biomass further supports circular economy approaches [13]. However, in many SSA contexts, insects are predominantly harvested from the wild, with estimates indicating that up to 92% are collected rather than farmed [30]. This reliance raises concerns regarding long-term sustainability, particularly given reported declines in insect availability in some regions [27]. Unregulated harvesting and limited monitoring frameworks may contribute to biodiversity loss and ecosystem imbalance, underscoring the need for the development of controlled farming systems and stronger regulatory oversight [17,30].

From an economic and social perspective, edible insects represent both an opportunity and a challenge. Insect collection, processing, and trade can support household income generation and local value chains, particularly in rural and low-income settings [6,25,30], and small-scale farming may offer a low-barrier livelihood strategy [26]. Nevertheless, the predominance of informal markets, seasonal supply, and limited infrastructure may constrain scalability and long-term viability [6,17]. Socially, entomophagy is deeply embedded in cultural traditions and widely accepted in many SSA communities, especially among older populations [11,18,23], representing a key advantage for dietary integration. However, religious beliefs, taboos, and perceptions of insects as unsafe, together with the influence of urbanization and westernized dietary patterns, may reduce acceptance among younger and more urban populations [6,11,28,34]. Overall, while edible insects hold significant potential to contribute to sustainable food systems in SSA, this potential is context-dependent and requires the alignment of production practices, regulatory frameworks, and socio-cultural acceptance [15,17].

This review has several limitations. First, it relies exclusively on PubMed and Google Scholar and does not include searches of African local journals nor the gray literature, which may be a risk of selection bias. Second, only eight countries are represented, resulting in limited geographic coverage. Although there are several studies from the DRC, other important research from Francophone regions (e.g., Senegal, Côte d’Ivoire) and East Africa is underrepresented. Third, the overall availability of data on edible insect consumption in SSA is limited, particularly with respect to regulatory frameworks governing their production and consumption. No subgroup analyses by insect species were performed to explore potential nutritional differences, as the primary aim of this review was to assess the general prevalence and frequency of entomophagy in the region; therefore, species-specific information was included only descriptively. Finally, the populations included in the selected studies are heterogeneous in terms of age, sex, and socioeconomic background, which may limit the generalizability of the findings. These limitations underscore the need for further research to support the large-scale implementation of insect-based dietary strategies.

Despite these constraints, this review provides valuable insights into the acceptability and potential of insect-based diets in SSA. Notably, there is a consistent pattern across studies indicating generally positive acceptance. Moreover, the inclusion of data from multiple countries enhances the diversity and representativeness of the findings. Overall, the evidence supports the potential of edible insects as a sustainable and nutritionally valuable alternative to conventional animal protein sources in low-income settings.

## 5. Conclusions

Edible insect consumption remains a longstanding and culturally embedded practice across sub-Saharan Africa, with beetles and termites among the most commonly consumed species, typically prepared using simple methods such as frying or boiling. Patterns of acceptance or rejection are largely shaped by cultural and traditional factors; however, the perceived nutritional value of insects is a primary driver sustaining their consumption. Current evidence suggests that edible insects constitute a rich source of high-quality protein and essential micronutrients, including calcium, zinc, and iron, highlighting their potential to contribute to addressing the triple burden of malnutrition. Nevertheless, important knowledge gaps persist, particularly regarding the standardization of nutritional profiles, safety, and long-term health impacts. Further research is therefore required to support the large-scale integration of insect-based foods into dietary strategies aimed at alleviating malnutrition in low-income settings, such as sub-Saharan Africa. Despite these limitations, entomophagy represents a promising, sustainable, and nutritionally valuable component of future food systems.

## Figures and Tables

**Table 1:** Main characteristics of studies on insect-based diets in sub-Saharan Africa, by country.

Reference	Country	Period	Study Design and Population	Main Objectives
[18]	Benin	2018	Qualitative N = 100	Knowledge of eating insects Desire to eat insects and associated factors Preferred insects Most popular processing methods
[19]	DRC	2022	Qualitative N = 60	Consumption Desire to eat insects Positive effects mentioned: Health risks Difficulties Reasons for not eating
[20]	DRC	2019	Qualitative N = 260	Consumption Preferred insects Influences on the preferences Seasonal availability Harvesting techniques Processing methods and preservation techniques
[21]	DRC	2020	Qualitative N = 520	Consumption Preferences and associated factors Harvesting techniques Processing and preservation
[22]	Kenya	2014	Cross-sectional within an RCT N = 47 children	Nutritional impact (Zn, fat)
[23]	Kenya	2021	Qualitative N = 211	Consumption and associated factors Tolerance Most common methods of preparing Perceived benefit of consuming Willingness to adopt Allergy or health
[24]	Kenya	2017	Field randomized experiment N = 432	Consumption and associated factors Positive emotions
[25]	Liberia	2020	Qualitative N = 255	Consumption Risks Barriers to obtaining insects
[26]	Liberia	2020	Field intervention Pre-post intervention (no control group) N = 16	Consumption Processing methods Most acceptability Reject consumption reasons Challenges for farming
[27]	Madagascar	2020	Series of cases N = 216	Preferred insects Reasons for consuming Reasons for not harvesting Protein and micronutrient intake
[11]	South Africa	2019	Qualitative	Consumption and associated factors Preferred insects Reasons for consuming Reasons for not consuming Processing methods
[28]	Tanzania	2016	Qualitative N = 50	Consumption Reasons for consumption Risks Beliefs Processing methods Trade
[6]	Zimbabwe	2021	Cross-sectional N = 304 households Focus group: N = 19	Consumption and associated factors Reasons for non-consumption Harvesting Trading Processing methods
[29]	South Africa	-	Review	Protein and mineral and trace elements Toxic and harmful antinutritional substances
[30]	SSA	-	Review	Risks (toxins and microbiological contamination) Production methods Protein and micronutrients

DRC: Democratic Republic of Congo; RCT: randomized controlled trial; SSA: sub-Saharan Africa.

## Data Availability

No new data were created or analyzed in this study. Data sharing is not applicable to this article.

## Abbreviations

The following abbreviations are used in this manuscript:

EFSA	European Food Safety Authority
FAO	Food and Agriculture Organization
SSA	Sub-Saharan Africa
WHO	World Health Organization

## Appendix A

**Table A1:** Detailed characteristics of studies on sustainable insect-based diets in sub-Saharan Africa, by country.

Reference	Country	Period	Study Design and Population	Methods	Consumption/Acceptability	Nutritional Impact
[18]	Benin	2018	Qualitative N = 100 - N = 50 Kétou (rural) 84% males - N = 50 Pobè (city) 48% males	Kétou: Semi-structured questionnaire: - Familiarity with insects as food - Attitude toward insects as diet - Ancestral knowledge transmission Pobè: Face-to-face interview using photos and species from market: - Preferences: certain edible insects	Knowledge of eating insects: 91% male/100% female Desire to eat insects: 45% male/50% women Transmission of tradition: - Elders (50% male/25% female) - Others: friends, in the market (Nigerian people selling), observing people collecting them and watching reports on television Positive feeling about consuming: 79% male/75% female 74% will offer them at parties Factors to eat insects: - 80% tradition - 70% test - ↓ % nutrition Preferred insects in urban region: beetle *Rhynchophorus phoenicis*, followed by crickets and termites. Most popular processing methods: Fry; grill; roast; raw (with spicy) Not insect farming in the country Market with Niger and Nigeria to import and export insects	-
[19]	Democratic Republic of Congo (DRC) (Kinshasa)	2022 Aug (dry season)	Qualitative N = 60 57% males Age: 14–73 years 53% high school/univ 63% employees 57% live in outskirts	Semi-structured interview: - Emotional aspects and habits related to insect consumption - Context in which people eat insects	98% like to eat insects (53% like and 45% like a lot) Positive effects mentioned: - Protein intake - Prevention of malnutrition - Help with food diversification 80% mentioned health risk: - Throat itching-excess crickets - Diarrhea-excessive caterpillars - Allergies 78% believed easy to obtain People who believed is difficult: - 62% difficult to obtain in market - 31% availability periodicity - 23% cost - 16% reasons related to people - 8% lack of transportation 95% intended to buy and eat edible insects the week of the interview. - ≥4 glasses (27%) - 2–3 glasses (42%) - 1–2 glasses (4%) - 1 glass (21%) - only half a glass (6%) 97% have always consumed them. Reasons for not eating: - taste (36%) - emotional reasons (32%) - personal beliefs (22%) - poor cooking (22%) - culture and habits (12%) - lack of knowledge (10%) - health reasons (5%)	-
[20]	DRC (South-Kivu Province)	2019	Qualitative N = 260 women and men Age: >18 years. N = 130 Kalehe N = 130 Idjwi Priority to people familiar with entomophagy.	Survey using 3 techniques: - structured oral interviews - direct observations - insect collection and taxonomy Data collected of edible insects: - diversity - host plants - seasonal availability - harvesting techniques - local processing methods - consumption preference	9 edible insects identified as a food source belonging to 9 families and five orders (wide diversity) Most preferred edible insects: - *R. differens* - *M. subhyalinus* Preferences are influenced by: - taste - shape - size - perceived nutritional value - color Seasonal availability (most of them in rainy season, September–May): - throughout the year - 9 months - 4 months Harvesting techniques depend on species. Three main techniques: - Harvesting - Handpicking - Light trapping Processing methods and preservation techniques depend on species and purposes: - Direct consumption - Preservation for further uses or market Most popular methods: boiled, fried, dry/fried, stewed, roasted or raw.	-
[21]	DRC (South-Kivu Province)	2020	Qualitative N = 520 women and men (N = 130 four territories) Age > 18 years All social classes. Priority to people familiar with entomophagy.	Field survey using three techniques: - structured oral interview - direct observations - insect collection Structured questionnaires: - commonly consumed insects - preferences and related factors - seasonal availability - host plants - harvesting techniques/timing - processing and preservation	Twenty-three edible insects identified as a food source belonging to 9 families. High diversity associated with the richness of the natural environment conditions of DRC. Preference: - *M. subhyalinus* - *R. phoenicis* - *R. differens* - *A. mellifera larvae* Preference linked to: - availability - ethnicity/cultures - palatability - seasonality - indigenous knowledge processing Caterpillars’ consumption common in DRC (Katanga, Bemba, Lamba tribes). Education crucial in increasing positive attitude towards consumption. Most valued for taste and size, but not for nutritional value or color. *A. domesticus*, *A. diaperinus* and *A. mellifera* larvae abundant all year, others depend on dry or rainy season. Not all edible insects need a host plant. Harvesting techniques: - trapping - collection - hand-picked Processing and preservation depend on the type and purpose: generally dried or dry/fried except honeybee.	-
[22]	Kenya	2014 Feb–Aug	Cross-sectional dietary intake within a randomized controlled trial (effect of Zn-fortified drinking water on Zn intake and bioavailability) N = 47 children Age: 2–3 years	Face to face interview with families and structured interviews. Data 24 h recalls of both days Data collected: - dietary intake - socio/demographic - anthropometry - blood sampling Two scenarios modeled: - FBR based on local foods - FBR based on local foods with house crickets	-	Zn would cease to be a problem nutrient when including house crickets to children’s diets. Population reference intake of Zn 89–121% in best scenario. Fat energy higher when house crickets included: 19–23% Three weekly servings of house crickets (portions of 46.5 g) could replace: - four weekly servings of small, whole fish with bones - two weekly servings of legumes
[23]	Kenya	2021	Qualitative N = 211 (55% female and 45% male) Communities known to consume beetle grubs Ten households per village Seven focus groups: N = 90	In person semi-structured questionnaire: - socio-demographics, knowledge, perceptions, and attitudes about beetle grubs - use beetle grubs as food-/feed-perceived benefits/problems - knowledge about grubs biology One to two focus groups discussions (10/15 participants each)	96.7% consumed ≥1 insect in prior 12 m - termites 94% - beetle grubs 39% Grubs consumption during harvesting season (rainy): daily 25%, weekly 34% Most common methods of preparing: - toasting (58%) - roasting (22%) Perceived benefit of consuming: - nutritiousness (28%) - tastiness (26%) Negative responses concerning consumption: - 24% lack knowledge grubs are edible - 5% fear of stigmatization 99% obtained from cattle dung Consumption and tolerance increase with age: - <35 years: 2.5–3.7% - >56 years: 51.9% Grubs consumption statistically higher in households headed by male. Consumption and education level: - 13% uneducated - 30% secondary education - 7% university graduates 78% used beetle grubs as animal feed. 58% believed grubs important in recycling nutrients 66% willingness to adopt beetle grubs farming if market and rearing protocols 100% no known allergy or health complication linked to eating the grubs. Focus group on eating beetle grubs: - culture in some counties - medicinal value, skin softening - more delicious than other insects. - no allergy or health complications	-
[24]	Kenya (Siaya and Machakos)	2017	Field randomized experiment N = 432	Consumer evaluation of the appropriateness of sensory attributes of cricket-flour-containing (CFC) buns. Participants randomly assigned to 3 treatment groups: - Control: general information on processing and safety of CFC buns - Treatment 1: general information and information about the benefits - Treatment 2: general information and information potential problems Evaluation CFC buns before and after tasting (Just-About-Right scale) Interview at homes: personal involvement, emotions, familiarity with insect-based foods and socio-economic characteristics.	90% familiar with and had consumed unprocessed edible insects in the past. 5% had consumed processed insect-based foods. More educated individuals and males perceived CFC buns more appropriate nutrition. Provided product information affected sensory evaluation. Actual buns tasting improved the convergence of sensory evaluation of the attributes towards the ideal level. This suggests the need to combine marketing with tasting during the introductory phase of the promotion. CFC buns elicited more positive feelings with little differences in the emotional profiles between the information treatments, which suggests general interest in the buns. “Curiosity” was the most common positive emotion.	-
[25]	Liberia	2020	Qualitative N = 255 Focus groups at 18 healthcare facilities with associated maternity waiting homes (MWHs) in 9 counties of Liberia. Participants: - N = 16 chiefs - N = 32 community leaders - N = 76 women reproductive age - N = 42 traditional birth attendants - N = 47 women at MWHs - N = 42 male partners	- Acceptability insect consumption by pregnant women in rural areas - Feasibility of insect farming for women staying at MWHs Focus groups, 12 questions: - food provision in MWHs - insect consumption practices - perceptions of insects - potential of using insects as an alternative source of income	Consumption of insects in pregnancy: - pregnant: primary consumers - termite, larvae, crickets, grasshoppers, … Perceived health impacts: + perceive health benefits + benefits for the baby + Palm weevill larva → “ringer neck” (baby receives sufficient nutrition) - Risk for diarrhea: prepare them improperly or eat too much at one time. Barriers to obtaining insects: - seasonal availability - difficulty of catching and maintaining edible insects Income/generating potential: marketability, particularly when available in the area (termite generates less money, but palm weevil, grasshopper and the “rubber disease” are very marketable)	-
[26]	Liberia (Bong county)	2020 Jul–Dec	Field intervention N = 16 Community health workers, public health consultants, pregnant women, opinion leaders and local leaders. Four MWH sites Two participants trained others during the project for a total of three new trainees.	One half-day workshop and trained: - start-up, maintain, and process edible insects as a sustainable food source for pregnant women - provided with starters for larvae storage facility construction - process palm weevil larvae into food powder, cubes, protein bars, pies, cakes, infants weaning feed Pre- and post-training knowledge assessment surveys Mixed-method project evaluation tracking sheets to document: - harvests - turnover cycles - barriers during project implementation - potential income generated through palm weevil production.	Preferred processing methods: - 50% grill; 25% fry; 25% boil - Others: roast and as a condiment Subproducts by larvae production: - 100% manure - 50% condiments - 25% firewood - 25% protein of soup Naama: - Most acceptability and consumption - All insects produced were consumed - No commercialization Phebe: - 50% consumed; 50% commercialized Janyea - Less interest in project; worst results Reject consumption: - Culture (indigenous tribes) - Not finding them appetizing 75% continue farming beyond the project. Challenges: implementation and sustainability (limited feed for larvae and lack of staff motivation). All sites reported easy-to-maintain farms if they have appropriate amounts of feed.	-
[27]	Madagascar	2020	Case study N = 216 - 66% male - 34% female 72% heads of household. Twelve villages of the region. Participation was voluntary and without remuneration.	Face-to-face interviews at households (preferentially to head household). - Quantitative and qualitive Data: insect harvest, buying and selling consumption Nutrition - food frequency tables for consumption for a whole year - nutritional composition (bibliographic research)	Two main reasons for consuming: - 88% good taste - 65% traditional food Considered by the local population as a full meat substitute: - Main dish with rice (92%) - Snack (7%) Insects vs. meat: - 52% choose meat - 37% choose insects - 11% meat and insects equally 82% would like to collect more insects. Reasons for not harvesting more: - lack of insects (79%) - labor constraints (12%) 81% have diminished trend of harvest insect quantities in recent years. Richer households: eat more meat and fish, but not less insects Poorer: less access to meat and fish, higher insects in protein consumption The more meat and fish proteins, the lower the share of insect protein	Insects: 10% of protein of rural population, same as pork and beef. Oct–Dec: highest harvesting (most consumed spp. only appear in these months). Oct–Nov: meat and fish diminish Preferred insects: - adult beetles (87%) - cicada (30%) - Coleoptera pupae (Abado 29%) - locusts (21%) Micronutrient intake: Adult scarab beetles (most consumed insect in region): iron 9.1 mg and zinc 8.8 mg/100 g edible portion.
[11]	South Africa (Limpopo and KwaZulu-Natal (KZN), provinces)	2019 KZN: Aug Limpopo: Dec	Qualitative Villages with insects consumption tradition. KwaZulu-Natal (KZN): - N = 500 - Five local municipalities 40% males/60% females Limpopo: - N = 122 - Four local municipalities in Vhembe 48% males/52% females	Face-to-face interviews. - Ways of assessing, processing and preparing, reasons of eating, consumption frequency, benefits and attitude towards eating. - List names of the insects consumed	Reasons for consuming (KZN/Limp): - Nutritional benefits (43%/66%) - Traditional beliefs (38%/21%) - Free and abundant of insects - Buffer during times of hardship Reasons for not (KZN): - Fear and discomfort (36%) - Western food adoption (27%) - Lack entomophagy knowledge 12% - Decrease the availability of edible insects in the wild (10%) Limpopo: - 3% religious belief KZN: - Termites (70%) the most preferred - 28% occasionally consumed - 64% consumed insects at least once - Factors that influence eating insects: age and educational background - All collected insects from the wild Limpopo: - Mopane worms (94%) most preferred - 95% occasionally consumed insects - 98% consumed insects at least once - Factors influence in choosing to eat insects: employment and gender - Almost half of respondents bought insects from towns Notable decline in entomophagy, particularly in KZN, related with: - Occidental acculturation - Discomfort associated with eating - Declining insects’ availability	Prepared by women using different methods (fried, boiled, roasted, sun-dried, or as a relish). - 62% preferred sun/dried insects in Limpopo - 88% preferred fried or roasted insects in KZN Cooking methods improve the sensory quality of edible insects. Edible insect flour should be incorporated in food products (biscuits, bread, energy bars, cereal, and cookies) to promote acceptability.
[28]	Tanzania (Muleba and Kagera)	2016 March to May	Qualitative N = 25 males N = 26 females (21–60 years) Three female and 7 male key informants among them	Face-to-face interviews about *Ruspolia differens* (senene): - perceptions, cultures, beliefs - indigenous technologies - harvest, processing, preserving - shelf life - traditions - prices - nutrition knowledge Observations in homestead, farms and wild fields, about: - harvesting - cooking - traditional processing Samples from fields and markets for identification and inventory	Five culturally significant forms of senene based on appearance and behavior of each. Varieties taste different; the tastiest was mwanamwana. Senene warm has decreased because of climate change Most natives believe senene is a God’s gift from heaven. Reasons for consumption: - 44% traditional delicious - 41% source of nutrients - 15% tasty nature as multipurpose sauce Considered a traditional delicacy, served at weddings, a symbol of respect/acceptance to the family. Eating it raw—prohibited (92% never eaten raw). It is associated with: - 52% stomachache - 48% diarrhea Use in preparation of: - Snacks - Rat bait - Chicken and fish feed Many African questioned taboos (man monopolizes insects as food): - Pregnant women prohibited to eat them because of malformation in babies - Infants and children would be unable to speak if they consume it No medicinal beliefs. Thought to be aphrodisiac. No allergies reported. Common to harvest at home under bright light. Obtention: - 43% market - 31% wild collections - 18% setting traps - 8% gifts from relatives Processing methods: increase palatability, safety and for preservation purposes. - Deep frying (preferred method) - Smoking (preferred preservation methods, up to 12 months) - Boling - Toasting - Sun drying Trade: - Men: commercial collection - Women: cleaning and processing - 43% obtain from local traders - Smoked, sundried and toasted not commonly found at market - Price of processed senene twice more expensive than fresh senene (laborious process)	Use of *R. differens* in enriching complementary foods for the contribution of reduction in stunting rate: - 32% boiled banana - 52% cereal porridge Despite taboos, most respondents reacted positively to include it in infants’ and children’s diets.
[6]	Zimbabwe (Gwanda)	2021	Cross-sectional mixed study. N = 304 households Focus group: N = 19 (in four selected wards)	Quantitative data: structured Q (household interview). - Edible insects’ value chain - Mopane worm value chain - Mopane worm harvesting, processing, consumption, trading Qualitative data: focus groups Knowledge, attitudes and practice on mopane worm value chain	Mopane worms most consumed—75% Termites (*Macrotermes* spp.)—7% 67% participate in the mopane worm chain. Main consumers: formally employed Harvesters/processors: 33% Pentecostal Consumers: 40% Protestant. Value chain dominated by: - 36% married participants - 27% single/never married - Only primary education Principal reason for non-consumption: religion/churches prohibition (not harvesting, consuming and trading). 17% likelihood for female-headed household to participate in value chain. Increasing 1 member household size increases 5% participation in value chain 73% harvest for both consumption and trade. Harvesting done by: - 30% whole family - 22% mothers only - 16% mother and children - 12% mother and father Harvesting more desirable during the fifth instar (minimal effort needed for degutting). Main methods of harvesting: picking from trees and from the ground. Trading: - 35% informal markets - 5% formal markets Processing methods: - Degutting - Boiling - Roasting - Sun-drying Limited diversity. Most common for relish to thick maize porridge.	Consumption is anticipated to have an effect on the overall food security as it provides nutrients and income to value chain participants. Fortification of maize porridge by mopane worm is key for bioaccessibility of important nutrients and contributing to RDA of children and adults. But for an extended use of mopane worm flour it is necessary to improve functional characteristics and sensory qualities it imparts to food stuff. This will facilitate utilization and provide more diversity to local diets.
[29]	South Africa	-	Review	Aimed to provide comprehensive information on availability of iron, zinc, copper, and manganese from selected edible insects, functions, and deficiencies in both humans and animals	-	Insects are mainly rich in protein and amino acids, but also in mineral and trace elements. The high protein content of insects is associated with improving the uptake of microminerals (Fe, Mn, Cu and Zn). - Fe and Zn are in non-hemoglobin forms (similar to that in plants) attached to proteins - Mopane worm (Gonimbrasia belina larvae) contains higher iron levels (51.05 mg/100 g) than meat from livestock - Higher iron and calcium contents compared to beef, pork, chicken, and other expensive protein - Significant variation in trace element contents (Fe levels between different species) Insects rich in trace elements: beetles, caterpillars, ants, grasshoppers, locusts, crickets, stinkbugs, termites, flies, and cockroaches. Extremely high Mn content: termites, mopane worms, house crickets, yellow mealworms, and locusts. Dietary supplementation of edible insects could help reduce Fe and Zn deficiency faced many marginalized households (children and pregnant women in rural communities) Indigenous edible insects (termites, worms, bugs, locusts, and grasshoppers) in Southern Africa could potentially help alleviate disease caused by a deficiency in trace elements. Presence of toxic and harmful antinutritional substances found in various insects (phytate, oxalate, and tannins); some contain high quantities of heavy metals.
[30]	SSA	-	Review	Study-available data on edible caterpillars in SSA regarding both ecology and rearing.	The sanitary quality of caterpillars collected from the natural environment raises some public health concerns (difficult to control the environment in which they are found, the level of toxins transferred from the host plants on which they feed, and possible risks of microbiological contamination). Harvested edible caterpillars also contribute toward improving the economy of local communities by marketing part of their harvest. Three possibilities for obtaining edible insects: - 92% harvested from the wild - 6% semi-domesticated - 2% farmed These production methods represent huge potential for the sustainable supply of edible insects, reducing negative impacts of wild harvesting on the natural environment	Edible caterpillars’ protein levels (28 g/100 g) vs. chicken meat (21 g/100 g). Energy intake of caterpillars (370 kcal/100 g) vs. pork (416 kcal/100 g). 100 g portion of insects is not enough to ensure the daily vitamin needs for humans (e.g., A and C); they contribute different vitamins (depending on species) and minerals. Their bioavailability provides a means of combating malnutrition in Africa and preventing metabolic diseases. Sometimes mixed with flour to prepare porridge for breakfast to combat malnutrition in children, frail people, and pregnant women. The digestibility of insect proteins is comparable to that of casein or soy proteins (77–98%). Digestibility improved by eliminating the rigid chitin-rich exoskeleton reduces the digestibility of their crude proteins. Particularly rich in lysine and threonine, sometimes deficient in methionine and cysteine. 100 g portion of Imbrasia truncata or Imbrasia epimethea caterpillars covered 30.6% and 32.3% of the required protein intake of a 75 kg adult. Rich in mono- and, even, polyunsaturated fatty acids, mainly linolenic and linoleic acids. Fe and Zn levels are similar or higher than conventional farm animals. Vitamin content of insects could be altered if they are raised on vitamin-rich substrates. Caterpillars are rich in water-soluble vitamins. Data available on vitamins varies across publications. These are attributed to the species used, analytical methods, and insect diet.

CFC: cricket-flour-containing; FBR: food-based recommendations; KZN: KwaZulu-Natal; MWHs: maternity waiting homes; RDA: recommended dietary allowance; SSA: sub-Saharan Africa; +: positive health impact; -: negative health impact.
